# A giant posterior mediastinal malignant peripheral nerve sheath tumor and benign neurofibroma in body surface: a case report

**DOI:** 10.1186/s12893-021-01122-5

**Published:** 2021-03-10

**Authors:** Yan Zhang, Hongfei Cai, Guangchao Lv, Yang Li

**Affiliations:** Department of Thoracic Surgery, The First Hospital of Jilin University, 71 Xinmin Street, Changchun, 130021 Jilin China

**Keywords:** Mediastinal tumor, Malignant peripheral nerve sheath tumor, Neurofibroma, Neurofibromatosis

## Abstract

**Background:**

Neurofibromatosis comprises neurofibromatosis type 1 (NF1) and type 2 (NF2). Major tumor type of NF1 are neurofibroma recognized as benign peripheral nerve tumor, malignant peripheral nerve sheath tumor (MPNST), and glioma.

**Case presentation:**

We report a woman with a special condition, whose tumors in body surfaces were benign neurofibroma and tumors in posterior mediastinum are MPNST. The chest-enhanced CT suggested a round soft tissue density in posteriormediastium. The diagnosis was established by pathology and immunohistochemistry. A single-stage thoracoscopic mediastinal mass resection was performed. The whole operation went smoothly and the CT scan of lungs did not show relapse of tumor three months later.

**Conclusions:**

The appearance of neurofibroma should draw particular attention to the possibility of developing MPNST. More careful imaging examinations should be carried out, and pathological examination could diagnose it.

## Background

Neurofibromatosis is a autosomal dominant disease that mainly implicates nervous system and causes tumor with distinct predisposition [1]. According to the classification of the National Institute of Health (NIH) in 1988, neurofibromatosis comprises neurofibromatosis type 1 (NF1) and type 2 (NF2). Major tumor type of NF1 are neurofibroma recognized as benign peripheral nerve tumor, malignant peripheral nerve sheath tumor (MPNST), and glioma [1–4]. NF2 includes bilateral vestibular nerve schwannomas as the most common tumor type, meningiomas, and ependymomas [1, 5].

**Fig. 1:**
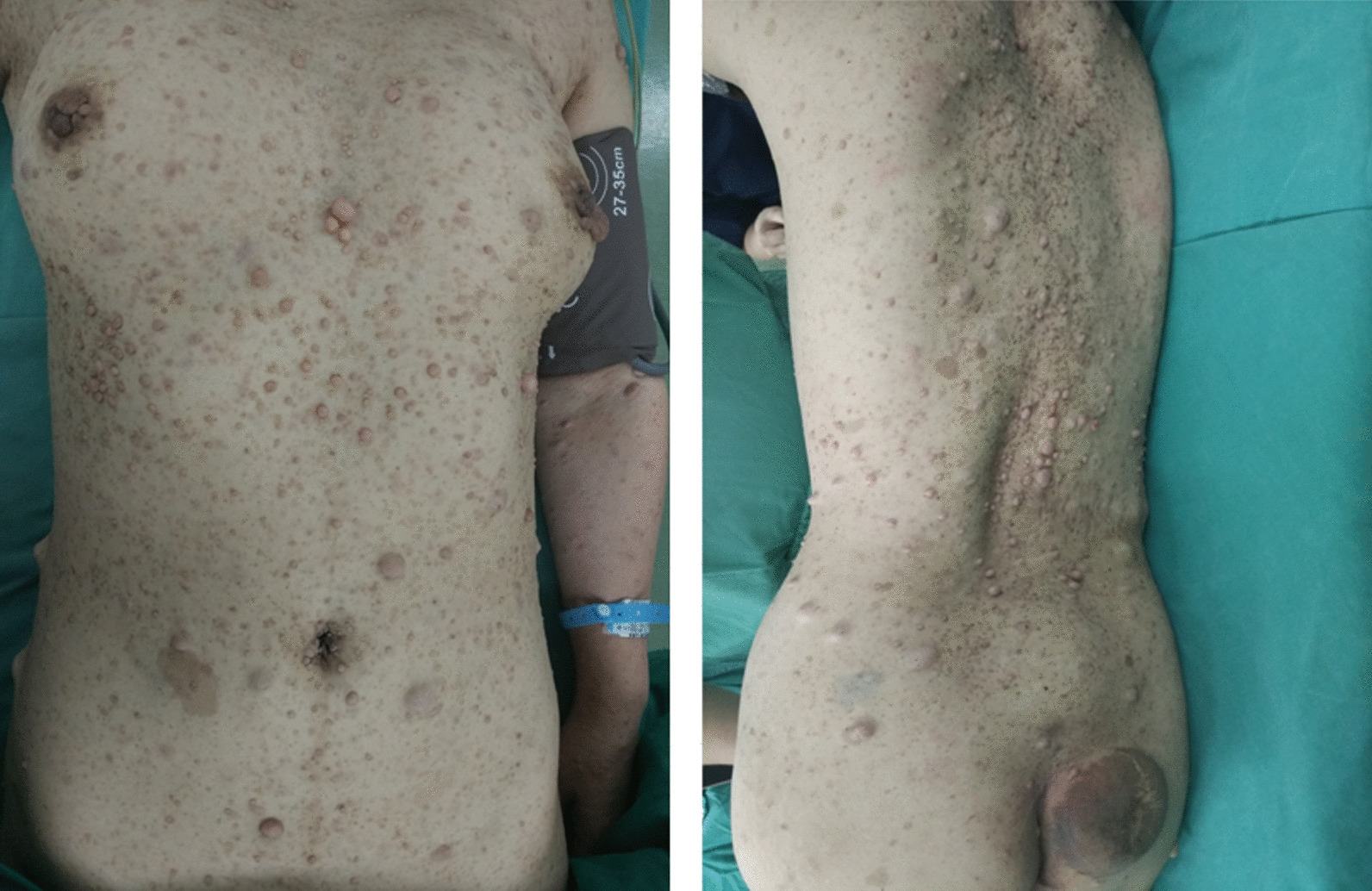
Photograph of neurofibroma in body surface

From clinical experience, neurocutaneous tumors rarely associated with esophageal neurogenic tumors and related symptoms. The patient reported in this case is a 50-year-old woman with a special condition, whose tumors in body surfaces were benign neurofibroma (Fig. 1) and tumors in posterior mediastinum are MPNST. Despite neurofibroma and nerve sheath tumor are both Schwann cell-derived tumors, their tumor characteristics and clinical manifestations are significantly different.

## Case presentation

This study was conducted under approval of the Ethics Committee of First Hospital of Jilin University, and the protocol was accorded with its standards.

**Fig. 2:**
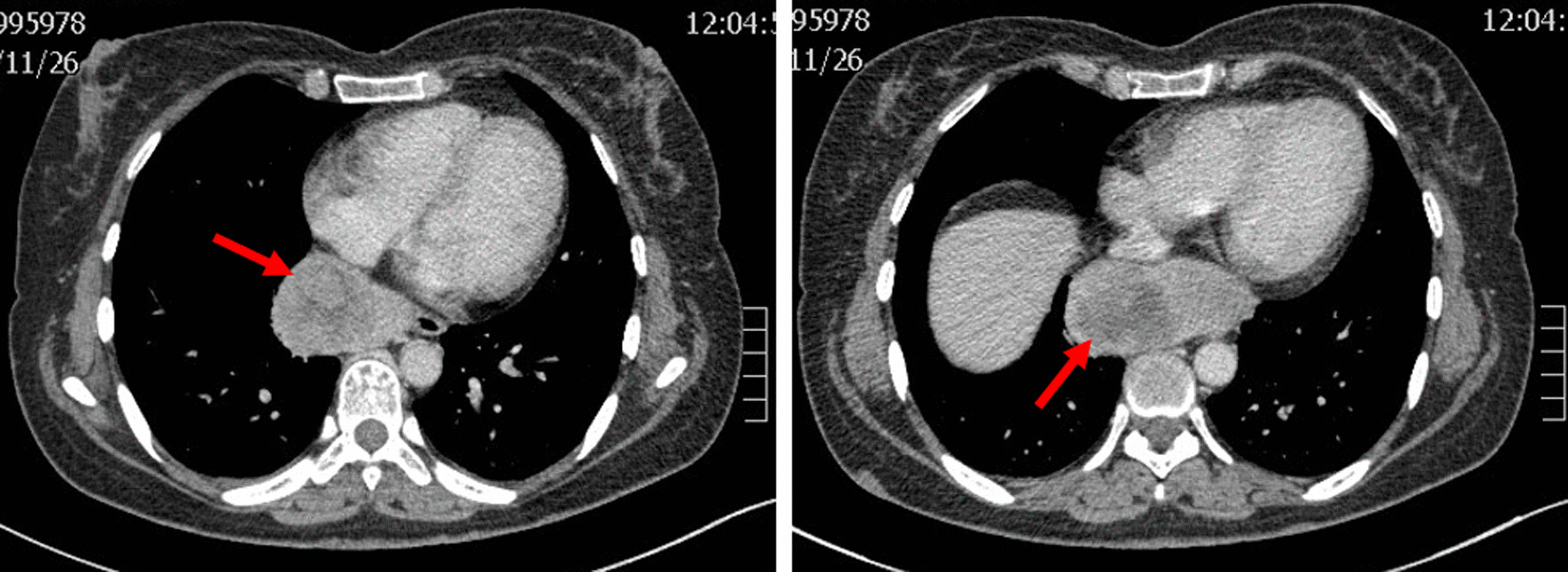
Chest enhanced CT showing a round soft tissue density sized 8.4 × 4.0 cm (red arrow)

This patient was a 50-year-old woman admitted to our department due to a posterior mediastinal mass detected by computed tomography (CT) scan 7 days ago. The symptom of oppression in chest did not improve after anti-inflammatory treatment. At admission, her blood pressure, pulse rate, respiratory rate and body temperature were all in the normal range. She had no cough and expectoration, no fever, no significant chest and back pain, and no muscle weakness. She had no hypertension, heart disease, diabetes, any infectious disease or drug allergy history. At the age of 15 years old, she was diagnosed of multiple neurofibroma. At the age of 48 years old, she received the fibormectomy at a local hospital because of worsened symptoms of the left lower extremity. At day 1 after admission, the chest-enhanced CT revealed, a round soft tissue density sized 8.4 × 4.0 cm with unclear boundary with esophagus and heterogeneous enhancement in posteriormediastium, and multiple nodule-like soft tissue density shadows in subcutaneous chest and back (Fig. 2). Meglumine diatrizoate angiography of upper gastrointestinal tract revealed abnormal changes in the lower esophagus. Magnetic resonance imaging (MRI) revealed thoracic bone hyperplasia and disc deformation. Laboratory examination showed that the plasma level and urinary excretion of epinephrine, norepinephrine and dopamine were all in the normal range. The liver function and kidney function were normal.

**Fig. 3:**
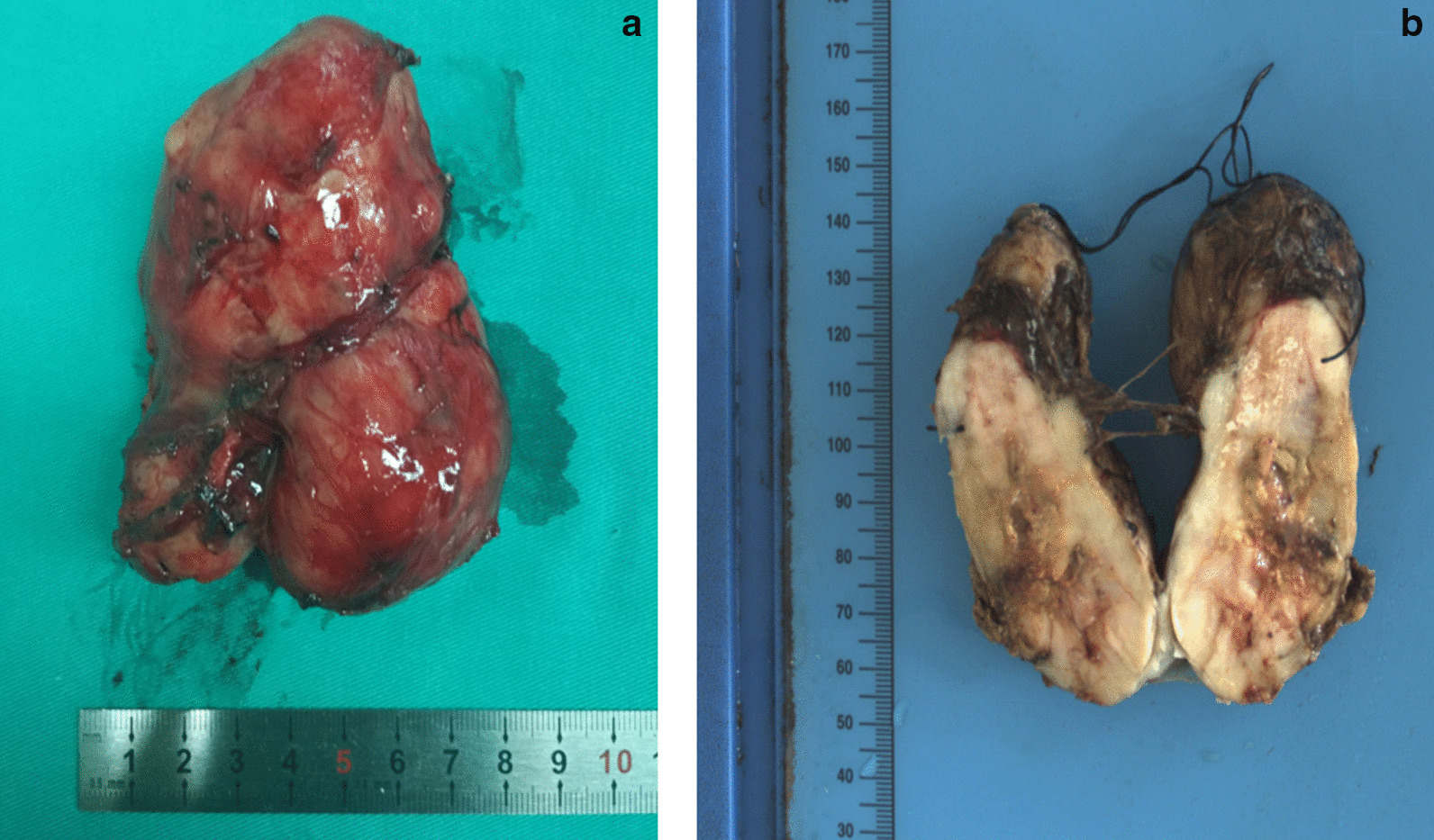
Intraoperative image showing a tumor with abundant blood supply and incomplete capsule (**a**), and postoperative image in histopathological examination (**b**)

Before operation, the patient was treated with intravenous fluid resuscitation for a week and prepared for a surgery. A single-stage thoracoscopic mediastinal mass resection was performed. The patient was placed in the right supine position and anesthetized by double lumen tracheal intubation. A about 1.5 cm-long incision in the 9th intercostal axillary midline was made and thoracic exploration was performed by thoracoscope first. Then, a standard 24 cm-long lateral incision in the 7th intercostal space on the left was made. After each layer was cut, switch to single lung ventilation. No effusion or adhesions in the thoracic cavity and no space occupying lesion in pulmonary lobe were observed. The lower mediastinal pleural bulged and a mass adhered to surrounding tissue could be felt behind the esophagus and in front of the aorta. No obvious enlarged lymph nodes were found in the mediastinum. Mediastinal mass was diagnosed intraoperatively. Then, the posterior mediastinal pleura was open. After fully dissociating mass with surrounding tissues, the mass was completely resected. Rapid intraoperative pathology reported posterior mediastinal tumor. The tumor was measured about 11 × 7 × 5 cm in size with abundant blood supply and incomplete capsule (Fig. 3a). The tumor location was marked by titanium clip and a drainage tube was placed and fixed in the 9th intercostal space of the left axillary midline. Finally, suture the chest wall layer by layer. A total of 1000 mL fluid without transfusion was given. The whole operation went smoothly and the patient was back to ward safely.

**Fig. 4:**
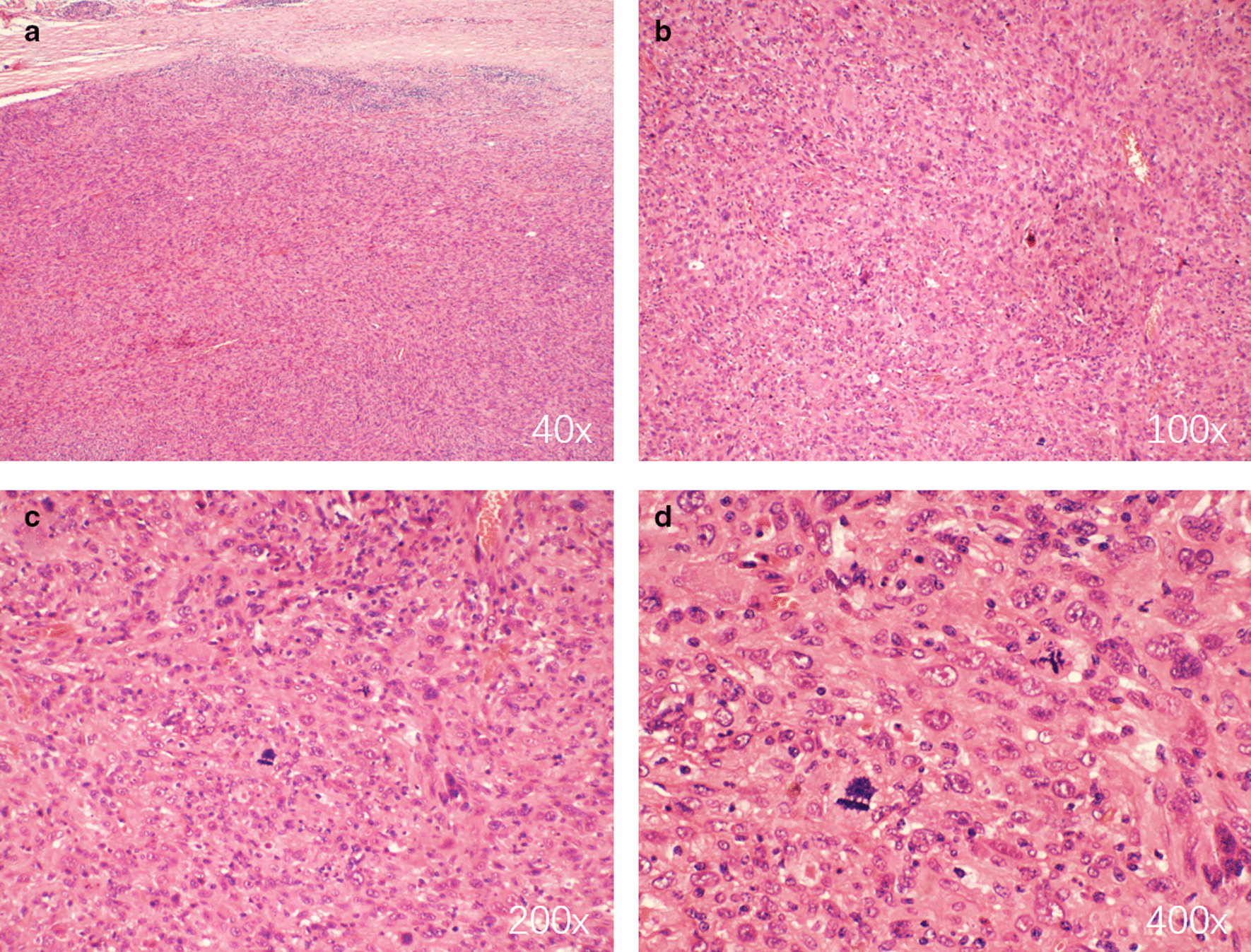
Hematoxylin–eosin (H&E) staining showed obvious tumor cell atypia and irregular nuclei > 10/10 HPF (High power field). **a** 40×; **b** 100×; **c** 200×; **d** 400×

**Fig. 5:**
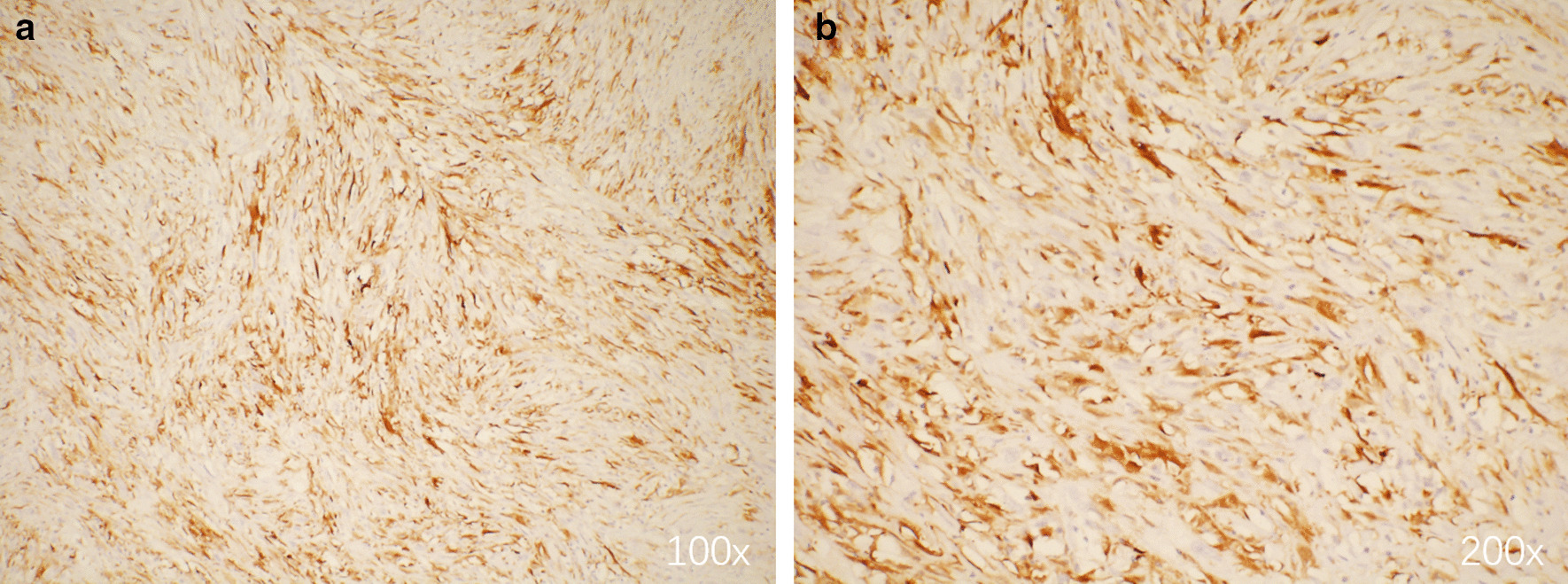
Immunohistochemistry staining of S-100 showing positive staining. **a** 100×; **b** 200×

**Fig. 6:**
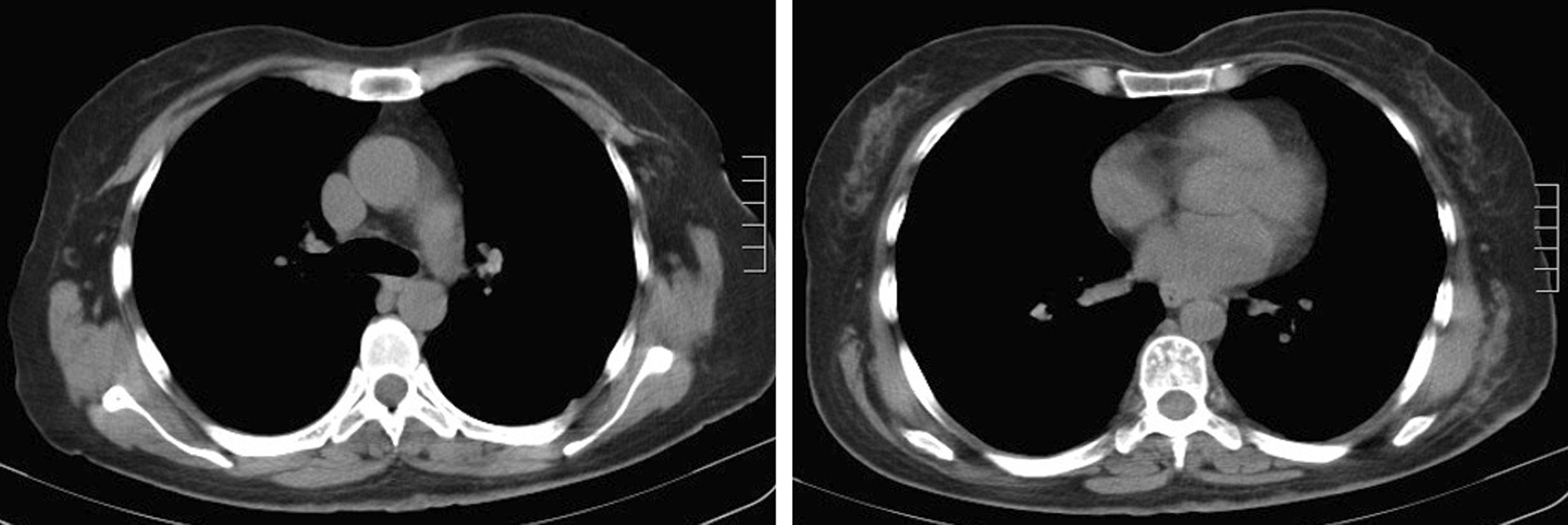
The CT scan of lungs did not show relapse of tumor after 3 months

Postoperative histopathological examination showed the tumor was MPNST and measured 9.0 × 7.0 × 4.8 cm in size (Fig. 3b). Hematoxylin–eosin (H&E) staining showed obvious tumor cell atypia and irregular nuclei > 10/10 HPF (High power field) (Fig. 4). Immunohistochemistry revealed CD34(−), Desmin(−), Ki-67(+ 30%), SMA( +), Vimentin( +), S-100( +), CD99( +), Bcl-2(−), CD117(−), EMA( +), and Calretinin( +) (Fig. 5). Moreover, histopathological examination showed the mass in the body surface was benign neurofibroma and immunohistochemistry revealed Ki-67(+ 1%) and S-100( +). Three months later, the CT scan of lungs did not show relapse of tumor (Fig. 6).

## Discussion and conclusions

NF1 with an global prevalence of approximately 1 in 3000, is more common than NF2 with a birth incidence from 1 in 25,000 to 40,000 by estimate [1–5]. Patients with NF1 may have a higher risk of cancer, and a decrease in life expectancy of ~ 8 to 21 years, especially a higher mortality for those younger than 40 years old [2, 6, 7].

This inherited disease is strongly associated with gene *Nf1*. The result of Le et al*.* showed loss of *Nf1* in skin-derived neural progenitors is required but not sufficient to induce tumors and suggested an essential role for the tumor microenvironment in neurofibroma development [8].

The clinical manifestations of NF1 include milk coffee spot, multiple neurofibroma, neurological symptoms, bone damage and visceral damage. As is known, neurofibromas are benign peripheral nerve sheath tumors arising from Schwann cell progenitors, of which cutaneous neurofibromas (cNF) are the most common type. The appearance of cNF varies, including nodular masses, peduncular lesions and diffuse plaques. They can be single or multiple, and are localized rather than encapsulated with no clear association with myelinated nerves [9]. Generally, cNF start developing early in adolescence, and keep increasing in number through the whole adulthood, which rarely transform into malignancy [10]. Surgical treatment is the preferred treatment for neurofibroma.

As for histology, S100 + Schwann cells were observed in surficial neurofibroma and part of posterior mediastinal MPNST. The significant difference between Ki67 expression in both tumor (1% of neurofibroma *vs.* 30% of nerve sheath tumor) indicated the dramatic cell proliferation. The positive biomarkers of nerve sheath tumor showed a possibility of its metastasis. Neurofibromas are mixed, consisting of neoplastic Schwann cells and non-neoplastic elements [8]. Usually, the diverse cellular components are embedded in collagenous and myxoid extracellular matrix [9]. Researches on finding new diagnostic tool with high sensitivity for MPNST never stop, such as Hirbe et al. reported β-III-spectrin immunohistochemistry as a potential diagnostic tool [11]. Besides traditional H&E staining and immunohistochemistry, a methylation-based classification of benign and malignant peripheral nerve sheath tumors was put forward, suggesting that the application of methylation status examination may facilitate the diagnosis of MPNST distinguish from benign nerve sheath tumors [12].

MPNST is a rare neoplasm of the peripheral nervous system. Most of the tumors occur in young and middle-aged men, and most of them occur in limbs, scalp and neck [13]. Atypical neurofibroma (ANF), with pathologically increased variable cellularity, cytological atypia and fascicular growth patterns, was regarded as precursor lesions for MPNST [13]. Other researchers also reported some cases of MPNST in the mediastinum [14–16]. Taken together, the appearance of neurofibroma should draw particular attention to the possibility of developing MPNST. Once NF1 is diagnosed, more careful imaging examinations should be carried out and needle biopsy is a more confirmative approach if possible.

## Data Availability

Not applicable.

## Abbreviations

NF1: Neurofibromatosis type 1
NF2: Neurofibromatosis type 2
MPNST: Malignant peripheral nerve sheath tumor
CT: Computed tomography
MRI: Magnetic resonance imaging
